# Snakebites and Antisnake Venom Utilization in Ghana's Oti Region: A 6-Year Retrospective Study

**DOI:** 10.1155/2024/6692421

**Published:** 2024-08-06

**Authors:** Courage Edem Ketor, Charles Kwaku Benneh, Kofi Boamah Mensah, Emmanuel Sarkodie, Adelaide Mensah, Samuel Owusu Somuah, Selorm Akakpo, Kwame Ohene Buabeng

**Affiliations:** ^1^ Pharmacy Department Jasikan District Hospital Ghana Health Service, Jasikan, Ghana; ^2^ Department of Pharmacy Practice School of Pharmacy University of Health and Allied Sciences, Ho, Ghana; ^3^ Department of Clinical Pharmacy and Pharmacy Practice Ulster University, Coleraine, UK; ^4^ Department of Pharmacy Practice Faculty of Pharmacy and Pharmaceutical Sciences College of Health Sciences Kwame Nkrumah University of Science and Technology, Kumasi, Ghana; ^5^ University Hospital Kwame Nkrumah University of Science and Technology, Kumasi, Ghana; ^6^ Department of Pharmaceutics School of Pharmacy University of Health and Allied Sciences, Ho, Ghana; ^7^ Pharmacy Department Ho Teaching Hospital, Ho, Ghana

**Keywords:** antivenom, neglected tropical diseases, snakebite

## Abstract

**Background:** Snakebite is a global environmental and occupational hazard and a significant public health threat. In rural areas, snakebite cases often go unreported and undocumented due to the lack of access to well-structured healthcare facilities/infrastructure. In some cases, the need for antisnake venom (ASV) far outstrips supply, negatively affecting treatment outcomes. This study, therefore, assessed the epidemiological characteristics of snakebite cases, their management, and how antivenoms are utilised at the selected hospital in the Jasikan District Hospital.

**Methods:** A 6-year retrospective study using secondary data from antivenom return forms (pharmacy records), clinical records (patient folders), the District Health Information Management System-2 (DHIMS-2) database, and consulting room registers was carried out in selected hospitals in the Jasikan District, Oti, Ghana.

**Results:** The predominant symptom of snakebite was localised pain (71.4%). The snakebite commonly occurred at home (19%) and on farms (18%). Of the 98 snakebite cases, ASV was administered to 73 (74.5%) cases. Supportive treatment applied included prophylactic antitetanus immunoglobulin (ATS) (80.6%), prophylactic antibiotics (63%), corticosteroids (80.6%), and analgesics (63%). 95% (*n* = 94) of complete recoveries were recorded; three were discharged against medical advice, and one was mortality. The supply and use of antivenom were erratic throughout the months of high incidence, partly due to inconsistent availability at the Regional Medical Stores. The average ASV vials and hospital stay duration were 1.23 ± 0.86 vials and 2.67 ± 1.97 days, respectively. Although the peak of snakebites occurs in April, May, and June, the demand for antivenom in April and May exceeded supply.

**Conclusion:** The outcome of most snakebite case management was appropriate, irrespective of inadequate ASV supply in certain months. The erratic antivenom supply should be aligned with seasonal and facility-use patterns to enhance regional snakebite management.

## 1. Background

Snakebite envenoming is a significant public health issue in sub-Saharan Africa. Every year, an estimated 5.4 million individuals are bitten by snakes, with 1.8 to 2.7 million occurrences of envenomation [1]. In Africa, an annual incidence of 1 million snakebites has been projected, and this primarily occurs in sub-Sahara, producing 100,000 to 500,000 envenomations and mortality between 10,000 and 30,000 [2].

A significant proportion of snakebite cases go unreported and undocumented because most victims reside in rural areas where social facilities, including hospitals, are lacking [2–4]. In sub-Saharan Africa and other low- and middle-income countries, the actual burden of snakebites is hard to measure because most bites occur in rural communities where the first port of call is traditional healers instead of reporting to health centres, such as Community Health-Based Planning Services compound, and hospitals for appropriate care and documentation [5–7].

In 2019, an assessment of the incidence of snakebites in the Oti and Volta regions of Ghana demonstrated that Nkwanta North District in the Oti Region recorded the highest cases (16.8%), with the majority of the cases occurring in the rainy season [8]. However, epidemiological data, including the morbidity and mortality from snakebites in this region, are unknown.

The most common medically significant snake species in Ghana that require polyvalent use are the West African carpet viper (*Echis ocellatus*), black-necked spitting cobra (*Naja nigricollis*), and puff adder (*Bitis arietans*) [9].

The venomous bites occur mostly in the rainy and harvesting seasons among farmers, students, and individuals who work in the bush with case concentrations in the northern part of the Oti Region [8].

Gathering local epidemiological, sociodemographic, and clinical data on snakebites is essential to help health professionals and policymakers develop relevant strategies to improve the management of antivenom utilization and reduce morbidity and mortality. The study is aimed at assessing the epidemiological characteristics of snakebite cases, the clinical profile of snakebites, the management and outcome of treatment, and determining the supply rate and utilization of antivenom in Jasikan District Hospital over 6 years.

## 2. Methods

### 2.1. Study Design

This study was a retrospective descriptive cross-sectional review using secondary data generated from antivenom return forms (pharmacy records), clinical records (patient folders), the District Health Information Management System-2 (DHIMS-2) database, and consulting room registers. A structured data extraction tool was used for the data collection.

### 2.2. Conceptual Framework

**Figure 1:**
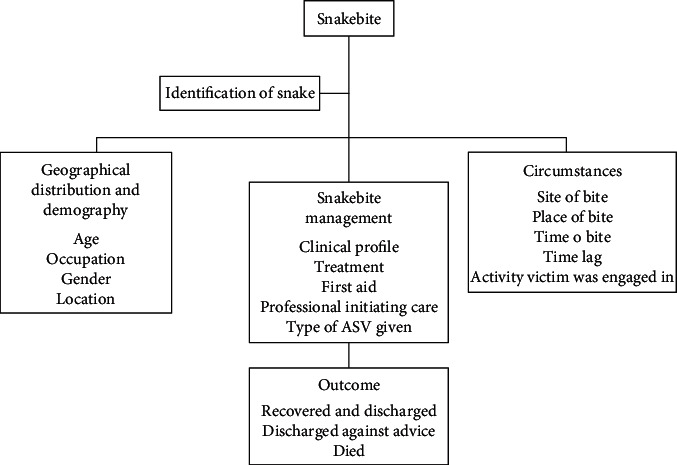
Conceptual framework for the study.

The management of snakebite involves identifying the type of snake responsible for envenomation, considering geographical distribution, demography, and bite circumstances. Early identification and appropriate therapy, including antivenom and other lifesaving procedures, significantly impact the treatment outcome at Jasikan District Hospital (Figure 1).

### 2.3. Study Site, Study Population, and Sampling Method

Jasikan District Hospital is a 53-bed public hospital run under the Ghana Health Service in the Oti Region of Ghana. The average daily outpatient attendance is 165. The study population comprised all available reported snakebite cases from 2014 to 2019. A total sampling technique was applied to select all eligible cases. Data were collected by a trained field officer and principal investigator from patient clinical records, pharmacy records, and antivenom return forms.

### 2.4. Ethical Issues

No formal ethical approval was required since snakebite is reported as part of the Integrated Disease Surveillance and Response (IDSR), and deidentified secondary data was used. However, the institutional review board of Jasikan District Hospital approved this study with reference number JDH/R/02 for secondary data to be analysed. In addition, the hospital authority provided anonymised and deidentified patient records from which we extracted the data for this study.

### 2.5. Statistical Analysis

Frequency tables were used to summarise data from the sociodemographic and clinical characteristics of patients. Statistical analyses were performed using SPSS Statistics 25 (IBM, United States) and SigmaPlot® Version 12.5 for Windows (Systat Software Inc., United States).

## 3. Results

### 3.1. Demography, Patient-Related Characteristics, and Distribution of Snakebites

**Figure 2:**
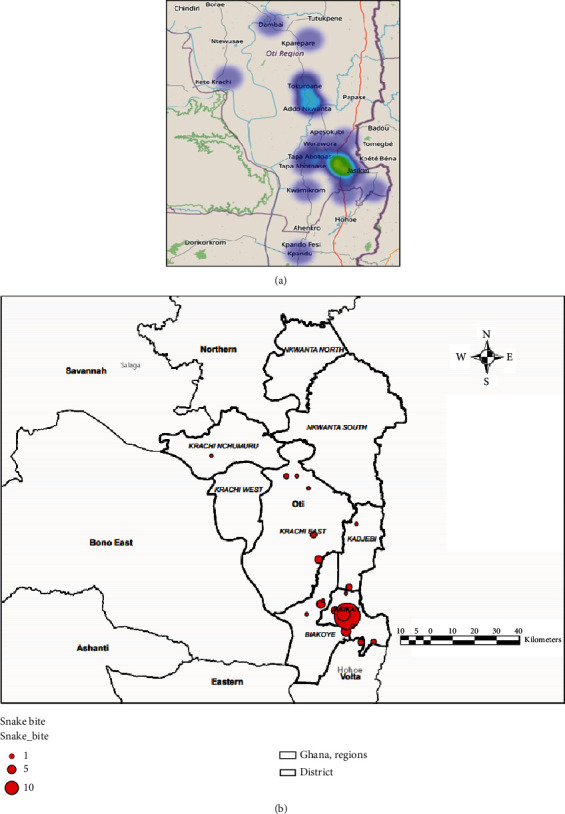
(a) Heat map of the location of case incidences managed at Jasikan Hospital. (b) The origin of bites and the distance to the treatment centre or access to antivenom in Jasikan District Hospital.

Out of 98 snakebites recorded within 6 years, the majority of the victims were males (59%, *n* = 58), older than 18 years (64.3%, *n* = 62), and students (49%, *n* = 48). The incidence of reported cases was unevenly distributed in the Krachi East (12.2%), Biakoye (18.4%), and Jasikan (48%) districts (Table 1). Due to the frequent shortage of antisnake venom (ASV) in the region, cases meant to be managed at district hospitals are often referred to the Jasikan District Hospital for care. The distribution of snakebite sources treated at the Jasikan District Hospital is shown in Figure 2.

Most cases were recorded within the Jasikan District, where the treatment centre is located. Snakebites from the northern part of the district (i.e., Dambai, Krachi, etc.) were managed at the Jasikan Hospital due to the nonavailability of antivenom along the continuum of care.

The most frequent bite sites (72.4%) were the lower limb (leg = 43, feet = 26, and knee = 2) (Table 2). The time of snakebites mainly (*n* = 94, 95.9%) occurred during the day, with a few cases (*n* = 4, 4.1%) occurring at dawn (12 a.m.–6 a.m.). The time lag between the snakebite and the initiation of clinical care was appreciable for most victims. Clinical care was initiated within 12 h for about 80% of victims, with about 2% receiving care in more than 72 h (Table 2).

Out of most of the clinical records reviewed, clinicians did not specifically state the identified snake implicated in the bite. The species and types of snakes were missing in the history of presenting complaints. Hence, the documented signs and symptoms of envenomation were therefore extracted for analysis.

### 3.2. Treatment Regimen, Trends in Supply, and Use of ASV

Antitetanus serum (ATS), steroids, antibiotics, infusions, and analgesics were mostly supportive care in managing snakebites in the facility aside from the antivenom (Tables 3 and 4). Paracetamol and diclofenac were commonly used analgesics, while penicillin and cephalosporin antibiotics were the most frequently used antibiotic classes. An ASV was not administered to about a fifth of the patients' cases reviewed (23.5%). The polyvalent ASV was mostly (74.5%) sourced from Regional Medical Stores and used for most reported cases, with a few (6.7%) sourced from the open market. Local or cytotoxic signs of envenomation comprised the majority of signs and symptoms observed. However, in about 80% of cases, no form of first aid was initiated (Table 3). In addition to the above, less than 15% of patients reported using some form of traditional medicine as first aid before reporting to a health facility which resulted in about 11% of patients being referred by a traditional healer (Table 3).

**Figure 3:**
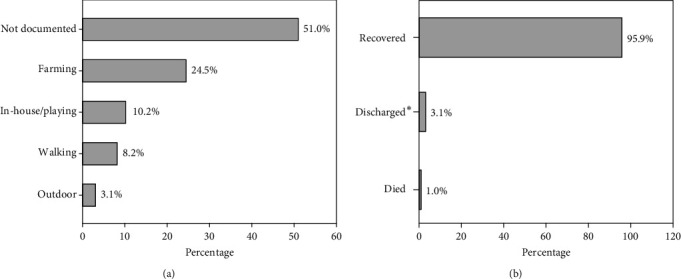
(a) Activities the victim was engaged in before the bite and (b) outcome of snakebites managed. ∗Discharged against medical advice.

The activities that victims were engaged in before the snakebites were not indicated; however, most of the documented cases were related to an outdoor activity compared to an in-door activity (35% vs. 10%) (Figure 3(a)). Almost all (95.9%) cases identified during the study period indicated complete recovery posttreatment, with only one patient (1%) dying and a minority of patients discharging themselves (3.1%) against medical advice (Figure 3(b)).

**Figure 4:**
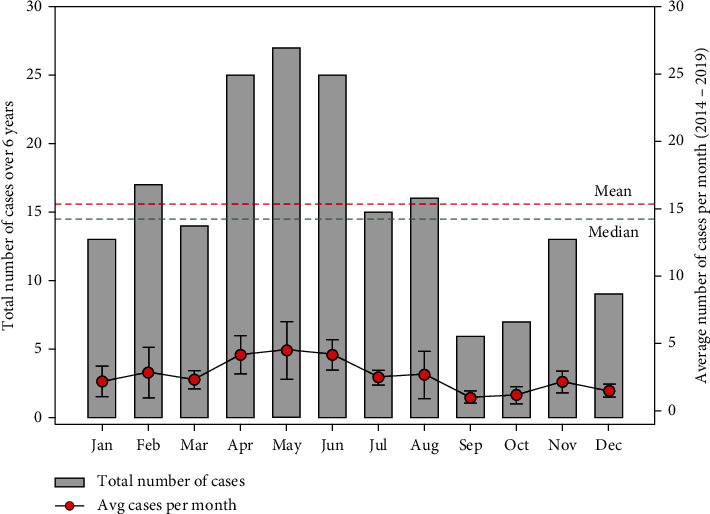
Trend of the monthly distribution of snakebite cases between 2014 and 2019, superimposed with the average number of cases per month over the same period.

**Figure 5:**
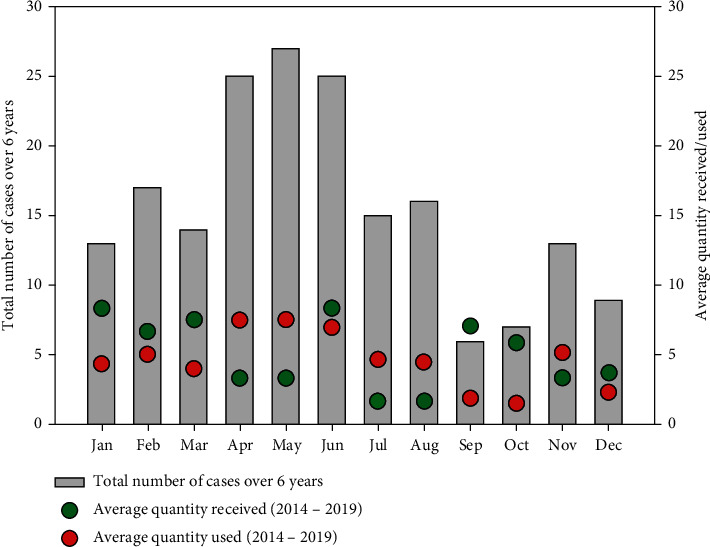
Trend chart of the supply of antivenom over the years under review. The bar graph shows the total number of reported snakebite cases in Jasikan between 2014 and 2019. The green and red dots represent the average number of antisnake venom serum received and used by the healthcare facilities in Jasikan, respectively.

The monthly distribution of snakebite cases over 6 years indicates higher case numbers for April, May, and June. In addition, February and August recorded case numbers greater than the mean cases (Figure 4). The supply and use of antivenom were erratic over the period evaluated partly due to inconsistent availability at the Regional Medical Stores. In April and May, the average demand for antivenom outstrips the hospital supply. However, in June, the supply and demand for antivenom were marginally comparable (Figure 5). In total, there was a deficit in the supply of antivenom for 5 months.

## 4. Discussion

The article presents an insight into the epidemiology, clinical features, and management of snakebite cases in the Oti Region of Ghana, a tropical country with a high burden of snakebite morbidity and mortality. We further highlight some challenges and gaps in the current health system, such as the lack of availability and accessibility of antivenom, the low awareness and utilization of first aid measures, and the influence of traditional medicine on health-seeking behaviour. In addition, some potential factors that may affect the incidence and outcome of snakebite cases, such as seasonal variation, occupational and recreational exposure, and the clinical presentation and response to treatment, are highlighted.

The majority of the victims were males, older than 18 years, and students. The mean age of patients presenting with snakebites in the Jasikan District Hospital was 26 years, similar to results from observations made by Punguyire et al. [10] and Mensah et al. [11]. Students and pupils recorded the highest number of cases (49%), followed by farmers (20%), which is contrary to an initial report by Yakubu, Abdul-Mumin, and Rivera [12], in which the majority of victims were farmers (46%), followed by students (29%). In rural areas, people of these ages often engage in open farming activities, increasing the risk of snakebites, a significant public health concern. The male gender was mostly affected by snakebites, recording 59% of all reported cases. This observation aligns with other reports [8, 12, 13]. This finding indicates that snakebite is a significant public health problem affecting the productive and vulnerable segments of the population, especially those engaged in outdoor activities such as farming, hunting, and schooling. Therefore, prioritizing these vulnerable groups for snakebite prevention education may be essential in reducing overall case incidences.

The incidence of reported cases was unevenly distributed in the Krachi East, Biakoye, and Jasikan districts, with the latter accounting for almost half of the cases. This suggests that there may be some geographical and ecological factors that influence the distribution and abundance of snakes and their habitats, as well as the exposure and risk of snakebite cases. Therefore, this finding implies that there is a need to conduct more studies on the epidemiology and ecology of snakebite cases in the region, as well as to implement more education and awareness campaigns on the prevention and protection measures for the population.

The peak period for snakebites occurred in the rainy season, confirming studies by Ceesay et al. [8] in the Volta Region of Ghana. Other researchers in north-east South Africa [14], Ethiopia [15], and Asia [16, 17] also bolstered the influence of rainfall on the increased incidence of snakebites. However, studies in Lokoja, Nigeria [18], and Kenya [19] recorded a high incidence of snakebites during the dry season. The peak occurrence of snakebites in the rainy season can be attributed to abundant food and water. The food attracts snakes to emerge from their hiding places during the dry season to hunt for food while at the same time coming into contact with humans going about farming or other activities during the rainy season. In situations where most of the bites occurred during the dry season, the researchers indicated that those snakebites occurred in the evening, where most of the victims in Africa slept outside due to the hot weather [18, 19].

Moreover, the location of the study site in Lokoja, Nigeria, was transitionally between the tropical and savannah regions, so it hardly records high incidences to produce enough sample size for the study. This geographical pattern of snakebite distribution across the globe needs to be considered by practitioners and community dwellers to minimize or avoid snakebites. The geographical distribution of cases that sourced antivenom from the Jasikan District Hospital suggests that antivenom's nonavailability makes victims travel in all directions to access care (see illustration, Figure 2). However, a minimal number of vials of ASV was used (Table 3), which is lower than the antivenom amount used in other regions of Ghana [12]. The ASV utilization per case was similar to the situation in other African countries and lower than the case in South Asia, where socioeconomic status is similar but the quality of antivenom used may be varied [18, 20].

One of the key findings is that most patients received supportive care in managing snakebite cases, aside from the antivenom, which was not administered to about a fifth of patients. This finding indicates that the hospital had a relatively well-equipped and staffed facility to provide basic care for snakebite victims, such as ATS, steroids, antibiotics, infusions, and analgesics. However, this finding also implies that the health facilities faced a shortage of antivenom, the most specific and effective treatment for snakebite envenomation. The polyvalent antivenom was mostly sourced from the Regional Medical Stores, which may have had inconsistent supply and distribution. In a few cases, antivenom that is obtained from the open market may raise concerns about the quality and safety of the product. Therefore, this finding suggests a need to improve the availability and accessibility of antivenom in hospitals and the region, as well as to ensure the quality and regulation of the product.

The time lag between the snakebite and the initiation of clinical care was appreciable for most victims, with about 80% of victims receiving care within 12 h and about 2% receiving care in more than 72 h. In a similar study by Punguyire et al. [10] in two district hospitals in Ghana (Bole District Hospital and Kintampo Municipal Hospital), about half the number of victims (49%) presented to the hospital between 4 and 12 h and only 5% of the respondents reported after 2–3 days. This situation could partly be due to the distance of their location, the nature of the road, access to transportation, financial difficulties, initial traditional or spiritual medical care, and disregard for the snakebites' effect. A similar length of time to access healthcare facilities has also been reported in South Asia [21]. Delayed hospital admission of snakebite patients in Ghana is coherent with findings from other African countries [22, 23] and Asian countries [21, 24]. However, this finding also implies that there may be some barriers and delays in seeking and reaching medical help, such as the distance, transportation, cost, and availability of health facilities and personnel. Therefore, identifying these barriers and proposing ways to mitigate their effects on care will improve overall care.

Another key finding is that most patients did not initiate any first aid, and some used traditional medicine before reporting to a health facility. This finding indicates a low awareness and utilization of first aid measures among the population, which may affect the outcome and prognosis of snakebite cases. The local or cytotoxic signs of envenomation comprised the majority of signs and symptoms observed, which may be exacerbated by the delay in seeking medical help. The utilization of hospitals by the majority of snakebite victims in Ghana may be due to the country's awareness and availability of snake antivenom [10]. Snakebite treatment-seeking behaviour in Jasikan District is quite similar to the findings of studies in other parts of Ghana [10, 25]. However, the treatment-seeking behaviour for snakebites is varied and complex, similar to some African countries [26] and South Asia [16].

Less than 15% of patients used some form of traditional medicine as first aid, and 11% of patients were referred by a traditional healer. In a study by Austin [27] at the Tamale Teaching Hospital in northern Ghana, he reported similar findings in 71% of cases presented directly from the community, but 2% of patients seeking help from traditional healers, as against 11% in Jasikan District Hospital. This finding suggests that traditional medicine plays a role in the health-seeking behaviour of snakebite victims, which may have positive or negative effects depending on the type and quality of the intervention. Therefore, there is a need to increase the awareness and education of the population on the appropriate first aid measures for snakebite cases, as well as to engage and collaborate with traditional healers to improve the referral and management of snakebite cases.

The snakebite management depends on the clinical syndrome presented due to the type of snake and the venom constitution. The WHO recommends the use of a corticosteroid as a routine premedication before administering antivenom [7]. This study however observed that corticosteroid (i.e., hydrocortisone injection) was used in only three out of four snake bite cases. Punguyire et al. [10] made similar observations in their study on the presentation, management, and outcome of snakebites in two district hospitals in Ghana, where 91% of cases had corticosteroids as part of the management. Of the cases studied, 63% were given antibiotics, 80.6% were given ATS, 63% were given analgesia, and 63% were given intravenous infusions. The use of ATS in all cases is encouraged, whereas the use of antibiotics prophylactically in all cases is not justified [28]. However, other studies have reported higher antibiotic consumption in managing snakebites [10, 12, 29]. The rational use of antibiotics to prevent secondary infections should be considered in managing snakebites to avoid antimicrobial resistance.

In terms of clinical care, it is encouraging to note that most clinicians who attended to snakebite cases were physician assistants who mostly work in rural areas. Snakebites are primarily associated with rural dwellings and farming activities; hence, cases are expected to be high in those areas. Since the majority of cases recovered satisfactorily (95%) with one recorded mortality (1%), it is recommended that antivenoms be supplied to health centres and physician assistants trained in managing snakebites as well. This will prevent patients from traveling several kilometers to the district hospitals to secure antivenom therapy.

The snakebites from the other parts of the district were managed at the Jasikan Hospital due to the nonavailability of antivenom along the continuum of care, which highlights a significant gap and disparity in the supply and distribution of antivenom in the region, which may affect the outcome and prognosis of snakebite cases. Therefore, there is a need to improve coordination and collaboration among the health facilities and stakeholders in the region and ensure the equitable and sustainable supply and distribution of antivenom.

The facility's supply and utilization of antivenom during the review period were erratic and unpredictable, which is coherent with findings from Dzenu, Agani, and Ayanore [25] and Habib et al. [30] in their study on the situations in Ghana and sub-Saharan Africa, respectively. Antivenom is classified as a program drug in the Ghana Health Service, and therefore, it is not licensed for purchase from hospitals or healthcare facilities. In this study, most antivenoms (93%) were supplied from the Regional Medical Stores and were accessible to patients. About 7% of cases had to be procured from the open market to fill up shortfalls in supply from the Regional Medical Stores. Therefore, the allocation of antivenom to health facilities should consider the facility's consumption and seasonal patterns and influences of demand. The scarcity of antivenom and frequent shortages resulted in victims traveling several kilometers to access care.

Most snakebites occur during outdoor activities, which indicates that snakebite is a major occupational and recreational hazard in the region, which may be influenced by environmental and ecological factors. The snakebite cases were higher in April, May, June, February, and August, which may correspond to the rainy season and the agricultural activities in the region. We identified April as the first month of the year where the average demand for ASV is not adequately matched by supply and is subsequently unresolved in May, highlighting the significant delay in reactive supply chain approaches. Improvements in supply–demand patterns in June are often not sustained, as shown by a significant deficit in the two subsequent months. This erratic nature of ASV supply delays prompts medical care for victims of snakebites.

Most patients recovered completely, with only one death and a few self-discharges. This finding suggests that the hospital had a relatively high success rate and quality of care for snakebite cases despite the challenges and limitations. Most patients recovered completely posttreatment, with only one patient dying and a minority of patients discharging themselves against medical advice. This finding suggests that the health system had a relatively good capacity and accessibility to provide prompt and effective care for snakebite victims despite the frequent shortage of antivenom in the region. However, this study may not be able to reveal some underreporting and underestimation of the snakebite cases and mortality in the region, as some patients may not seek or reach medical help or may die after discharge without being recorded. There is, therefore, a need to improve the surveillance and reporting of snakebite cases and outcomes in the region and to prevent and reduce the exposure and risk of snakebite cases.

Further studies are aimed at investigating and summarizing the complications of snakebites, as evidenced by laboratory results, the benefits of steroid use, and the seasonal distribution of snakebites, which will be beneficial to tailored symptomatic management of snakebites specific to the district. In addition, treatment protocols should be developed, adapted, and strictly adhered to improve patient outcomes and limit the unwarranted use of medications such as antibiotics.

## 5. Limitations

The retrospective nature of this study is its main limitation. This is because some important details have been lost because they were not documented in the medical records.

The study may have underestimated the burden and impact of snakebites in the region. The study relied on hospital records of snakebite cases, which may not capture the true incidence and mortality of snakebites in the region, as some victims may not seek or reach medical help or may die after discharge. Therefore, the study did not explore the sociocultural and economic factors that may influence the health-seeking behaviour and outcome of snakebite victims, such as the beliefs, attitudes, and practices of the population and the traditional healers, as well as the cost and affordability of antivenom and other medical services. Therefore, the study may have overlooked potential barriers and facilitators for improving the region's prevention and treatment of snakebites.

## 6. Conclusion

The study reveals the erratic nature of the antivenom supply, resulting in delays in medical care for snakebite victims. The study suggests that snakebite management can yield positive clinical outcomes despite challenges in the healthcare system and calls for antivenom availability at all primary care centres.

Further investigations into complications, benefits of treatment protocols, and matching seasonal snakebite patterns to ASV supply can contribute to more effective snakebite management in the region.

## Figures and Tables

**Table 1:** Demographic distribution of snakebites surveyed (*N* = 98).

**Variable**	**n**	**%**	**Mean (SD)**
Gender			
Male	58	59.2	
Female	40	41.8	
Age			
< 5 years	3	3.1	26.7 (16.5)
5–11 years	13	13.3	
12–18 years	19	19.4	
> 18 years	62	64.3	
Occupation			
Farming	20	20.4	
Student/pupil	48	49	
Formal work	9	9.2	
Business/trading	5	5.1	
Hairdressing	1	1.0	
Mason	1	1.0	
Hunter	1	1.0	
Unknown	13	13.3	
District/subdistrict			
Baika/Ayoma	2	2.0	
Biakoye	18	18.4	
Bodada	3	3.1	
Hohoe	2	2.0	
Jasikan sub	47	48	
Kadjebi	4	4.1	
Krachi East	12	12.2	
Nsuta	6	6.1	
Missing	4	4.1	

**Table 2:** Timing of snakebite and time lag between bite and clinical care (*N* = 98).

	**n**	**%**
Time of bite		
12 a.m.–6 a.m. (dawn)	4	4.1
6 p.m.–12 a.m. (night)	19	19.4
6 a.m.–12 p.m. (morning)	47	48.0
12 p.m.–6 p.m. (afternoon–evening)	28	28.6
Time lag before receiving clinical care		
0–12 h	78	79.6
12–24 h	3	3.1
24–48 h	10	10.2
48–72 h	5	5.1
> 72 h	2	2.0
Site of bite		
Upper limb		
Arm	5	5.1
Hand	9	9.2
Lower limb		
Feet	26	26.5
Leg	43	43.9
Knee	2	2.0
Not documented		
Not documented	8	8.2

**Table 3:** Clinical profile of snakebites managed.

	**n**	**%**
Type of envenomation (signs)		
Cardiovascular/haemorrhagic	3	3.1
Local/cytotoxic	70	71.4
Neuromuscular/neurotoxic	2	2.0
Neuromuscular & local	7	7.1
Cardiovascular & local	2	2.0
Neuromuscular & cardiovascular	1	1.1
None	13	13.3
First aids		
Traditional medicine	14	14.3
Incision/sucking	2	2.0
Black stone	1	1.0
None	79	80.6
Other∗	2	2.0
Reporting to care		
Directly from the community/house	82	83.7
Referred from a traditional healer	11	11.2
Referred from a health facility	5	5.1
Variable		Mean ± SD
Duration of hospital stay (days)		2.67 ± 1.97
The average number of vials of ASV used (1 vial = 10 mL)		1.23 ± 0.86

The ^∗^ represents an unknown injection from over-the-counter medicines seller.

**Table 4:** Management of snakebite and medications used (*N* = 98).

**Variable**	**n**	**%**
Treatment received		
Steroids (hydrocortisone injection)	76	77.6
Antitetanus serum	79	80.6
Antibiotics	62	63.3
Antisnake venom	73	74.5
Analgesic	62	63.3
Antifibrinolytic (tranexamic acid)	2	2.0
Intravenous infusion	62	63.3
Antiemetic	1	1.0
Diuretic	1	1.0
Category of professional initiating treatment		
Physician	36	36.7
Physician assistant	43	43.9
Nurse	19	19.4
Type of antivenom used		
Polyvalent	73	74.5
Monovalent	1	1.0
Both	1	1.0
Not given	23	23.5
Source of ASV		
Regional Medical Stores	70	93.3
Open market	5	6.7
Antibiotics^a^		
Amoxicillin ± clavulanic acid	42	42.9
Flucloxacillin	10	10.2
Benzylpenicillin	16	16.3
Cefuroxime	2	2.0
Metronidazole	2	2.0
Ampicillin	1	1.0
None	27	27.6
Analgesic^b^		
Oral paracetamol	27	27.6
Oral NSAID	28	28.6
Oral tramadol	3	3.1
Inj. tramadol	6	6.1
Inj. pethidine	4	4.1
Supp diclofenac	9	9.2
IVF^c^		
Multiple responses		Percent of cases
Sodium chloride 0.9% infusion	55	88.7
5% dextrose in 0.9% sodium chloride infusion	12	19.4
Dextrose infusion (5% or 10%)	11	17.7

^a^Details of antibiotics used.

^b^Details of analgesics used.

^c^Details of intravenous infusion (IVF).

## Data Availability

The datasets generated during and analysed during the current study are available from the corresponding author upon reasonable request.

## Nomenclature

ASV: antisnake venom
ATS: antitetanus serum
DHIMS: District Health Information Management System
NSAID: nonsteroidal anti-inflammatory drug
WHO: World Health Organization

## References

[1] WHO (2023). Snakebite envenoming (key facts). https://www.who.int/news-room/fact-sheets/detail/snakebite-envenoming.

[2] Chippaux J. P. (2011). Estimate of the burden of snakebites in sub-Saharan Africa: a meta-analytic approach. *Toxicon*.

[3] Chippaux J. P. (1998). Snakebites: appraisal of the global situation. *Bulletin of the World Health Organization*.

[4] HAI (2019). *HAI snakebite project in Zambia*.

[5] Mohapatra B., Warrell D. A., Suraweera W. (2011). Snakebite mortality in India: a nationally representative mortality survey. *PLoS Neglected Tropical Diseases*.

[6] Rahman R., Faiz M. A., Selim S. (2010). Annual incidence of Snake Bite in rural Bangladesh. *PLoS Neglected Tropical Diseases*.

[7] WHO (2010). *Guidelines for the prevention and clinical management of snakebite in Africa*.

[8] Ceesay B., Taal A., Kalisa M., Odikro M. A., Agbope D., Kenu E. (2021). Analysis of snakebite data in Volta and Oti regions, Ghana, 2019. *Pan African Medical Journal*.

[9] Gyawu V. B., Firempong C. K., Hamidu J. A. (2023). Production and evaluation of monovalent anti-snake immunoglobulins from chicken egg yolk using Ghanaian puff adder (Bitis arietans) venom: isolation, purification, and neutralization efficacy. *Toxicon*.

[10] Punguyire D., Baiden F., Nyuzaghl J. (2014). Presentation, management, and outcome of snake-bite in two district hospitals in Ghana. *Pan African Medical Journal*.

[11] Mensah E. K., Karikari K., Aikins M. (2016). Secondary analysis of snake bite data in the western region of Ghana: 2006- 2010. *Ghana Medical Journal*.

[12] Yakubu A.-S., Abdul-Mumin A., Rivera O. (2018). *Evaluation of management of snakebites in a teaching hospital in northern Ghana-a retrospective descriptive study*.

[13] Raina S., Raina S., Kaul R., Chander V., Jaryal A. (2014). Snakebite profile from a medical college in rural setting in the hills of Himachal Pradesh, India. *Indian Journal of Critical Care Medicine*.

[14] Wood D., Sartorius B., Hift R. (2016). Snakebite in north-eastern South Africa: clinical characteristics and risks for severity. *South African Family Practice*.

[15] Abdullahi A., Yusuf N., Debella A. (2022). Seasonal variation, treatment outcome, and its associated factors among the snakebite patients in Somali region, Ethiopia. *Frontiers in Public Health*.

[16] Pandey D. P., Thapa N. B. (2023). Analysis of News Media-Reported Snakebite Envenoming in Nepal during 2010-2022. *PLOS Neglected Tropical Diseases*.

[17] Pandey D. P. (2007). Epidemiology of snakebites based on field survey in Chitwan and Nawalparasi districts, Nepal. *Journal of Medical Toxicology*.

[18] Fadare J. O., Afolabi O. A. (2012). Management of snake bite in resource-challenged setting: a review of 18 months experience in a Nigerian hospital. *Journal of Clinical Medical Research*.

[19] Ochola F. O., Okumu M. O., Muchemi G. M., Mbaria J. M., Gikunju J. K. (2018). Epidemiology of snake bites in selected areas of Kenya. *Pan African Medical Journal*.

[20] Pandey D. P., Vohra R., Stalcup P., Shrestha B. R. (2016). A season of snakebite envenomation: presentation patterns, timing of care, anti-venom use, and case fatality rates from a hospital of southcentral Nepal. *Journal of Venom Research*.

[21] Pandey D. P., Subedi Pandey G., Sapkota S., Dangol D. R., Devkota N. R. (2023). Attitudes, knowledge and practices of traditional snakebite healers in Nepal: implications for prevention and control of snakebite. *Transactions of the Royal Society of Tropical Medicine and Hygiene*.

[22] Barnes K., Ngari C., Parkurito S. (2021). Delays, fears and training needs: perspectives of health workers on clinical management of snakebite revealed by a qualitative study in Kitui County, Kenya. *Toxicon: X*.

[23] Lang H. J., Amito J., Dünser M. W., Giera R., Towey R. (2020). Intensive-care management of snakebite victims in rural sub-Saharan Africa: an experience from Uganda. *Southern African Journal of Critical Care*.

[24] Pandey D. P., Shrestha B. R., Acharya K. P. (2023). A prospective study of snakebite in a tertiary care hospital in south-western Nepal. *Transactions of the Royal Society of Tropical Medicine and Hygiene*.

[25] Dzenu M. W., Agani A., Ayanore M. A. (2023). *Community and health system factors influencing snake envenomation management practices in three districts of Ghana: a qualitative inquiry from health stakeholders and snakebite victims*.

[26] Larson P. S., Ndemwa M., Thomas A. F. (2022). Snakebite victim profiles and treatment-seeking behaviors in two regions of Kenya: results from a health demographic surveillance system. *Tropical Medicine and Health*.

[27] Austin G.-A. (2018). *Retrospective study of snakebite cases at the Tamale Teaching Hospital*.

[28] Jorge M., Malaque C., Ribeiro L. (2004). Failure of chloramphenicol prophylaxis to reduce the frequency of abscess formation as a complication of envenoming by Bothrops snakes in Brazil: a double-blind randomized controlled trial. *Transactions of the Royal Society of Tropical Medicine and Hygiene*.

[29] Visser L., Kyei-Faried S., Belcher D. (2004). Protocol and monitoring to improve snake bite outcomes in rural Ghana. *Transactions of the Royal Society of Tropical Medicine and Hygiene*.

[30] Habib A. G., Musa B. M., Iliyasu G., Hamza M., Kuznik A., Chippaux J.-P. (2020). Challenges and prospects of snake antivenom supply in sub-Saharan Africa. *PLOS Neglected Tropical Diseases*.

